# CBAG: Conditional biomedical abstract generation

**DOI:** 10.1371/journal.pone.0253905

**Published:** 2021-07-06

**Authors:** Justin Sybrandt, Ilya Safro

**Affiliations:** 1 School of Computing, Clemson University, Clemson, SC, United States of America; 2 Department of Computer and Information Sciences, University of Delaware, Newark, DE, United States of America; Ulm University, GERMANY

## Abstract

Biomedical research papers often combine disjoint concepts in novel ways, such as when describing a newly discovered relationship between an understudied gene with an important disease. These concepts are often explicitly encoded as metadata keywords, such as the author-provided terms included with many documents in the MEDLINE database. While substantial recent work has addressed the problem of text generation in a more general context, applications, such as scientific writing assistants, or hypothesis generation systems, could benefit from the capacity to select the specific set of concepts that underpin a generated biomedical text. We propose a conditional language model following the transformer architecture. This model uses the “encoder stack” to encode concepts that a user wishes to discuss in the generated text. The “decoder stack” then follows the masked self-attention pattern to perform text generation, using both prior tokens as well as the encoded condition. We demonstrate that this approach provides significant control, while still producing reasonable biomedical text.

## Introduction

Scientific papers often combine a range of disconnected concepts in novel patterns, following the typical research strategies of many scientists [1]. Therefore, we anticipate that future applications, such as scientific writing assistants, will produce more usable results if they are informed of the user’s particular concepts of interest. This presents two challenges that we find to be unexplored in the modern text generation literature. Firstly, the number of concepts a user might wish to include is highly variable. Secondly, the range of concepts a user might wish to select from is large (tens of thousands). Therefore, we present the Conditional Biomedical Abstract Generation (CBAG) model, which enables controlled generation of biomedical abstracts.

While many transformer-based [2] Natural Language Processing (NLP) models have debuted in recent years, such as the popular BERT [3] and GPT/GPT-2 models [4, 5], as well as derivative models specialized for scientific text, including SciBERT [6] and BioBERT [7], there has been less work on conditional language modeling. The CTRL model [8], while enabling conditional text generation, does so by specifying a small fixed set of tokens that prefix an input sentence before applying the GPT-2 architecture. We find that this technique, while effective for applications like style transfer, where the number of “styles” is relatively small, is not expressive enough for conditioned generation of biomedical text. We find the same limitation with older conditional models, such as those designed for image captioning systems [9], that generate text given a single image encoding. More generalizable methods, such as those produced by variational auto-encoders [10], can capture rich latent language semantics, but cannot straightforwardly encode domain-based information, such as a set of keywords one wishes to include in the output text.

The CBAG model is a transformer featuring a shallow encoder stack to encode qualities of the condition and a deep decoder stack to produce a high quality language model. We train this model using semi-supervised multi-task generative pre-training, wherein to minimize our proposed objective function, the model must predict successive tokens, parts of speech, dependency tags, as well as entity labels. We train this model using over 20-million biomedical records provided by the National Library of Medicine (NLM) through the MEDLINE database. Each record consists of a title, abstract, publication year, and an optional set of author-provided keywords. Text processing and annotations are provided by a biomedical NLP model trained on the “BIONLP13CG” BioCreative training set [11]. This pre-trained domain-specific model allows the CBAG model to apply the knowledge gain from the relatively small human-annotated dataset to a much larger set of unstructured text from MEDLINE. We train the proposed model by sampling textual windows from within MEDLINE abstracts. The publication date, and any author-supplied Medical Subject Headings (MeSH terms, a set of biomedical keywords and phrases) form the condition. Windows are split into subword units using the unigram subword-regularization [12]. Using masked-self attention, we train the model to predict each subword *i* + 1 using only the condition and tokens 1, …, *i*.

We compare the CBAG model to two versions of GPT-2. First, we consider the 1.5B parameter version of the model, but due to technical limitation we do not finetune this model for abstract generation. However, prior work has identified that the GPT-2 “huge” model can, without finetuning, succeed in a range of specific tasks across domains, such as language translation, question answering, and commonsense reasoning [5], as well as function as a general-purpose knowledge base [13]. We secondly consider a smaller finetuned version of GPT-2 (124-million parameters) for abstract generation. Across all models, we compare generation quality through *n*-gram recall metrics.

We evaluate computer-generated abstracts based on their ability to produce relevant *n*-grams that occur in the human-written abstract associated with the input title. We leverage a range of Natural Language Generation (NLG) metrics [14], such as Bleu, METEOR, ROUGE-L and CIDEr, including a version of CIDEr that omits input *n*-grams from consideration. We find that even though CBAG was only trained on biomedical abstracts, a much smaller dataset than the GPT-2 models were initialized on, it performs similarly to the GPT-2 finetuned model on *n*-gram recall. However, we also demonstrate qualitatively that the CBAG model is capable of generating highly controlled textual output by speficiying different conditions to the same input text.

The remainder of this paper is organized as follows: In Background we provide an overview of language modeling and the transformer architecture. In Multi-Conditional Language Model we describe the methodology behind the CBAG model, which specializes the transformer architecture for generating biomedical abstracts. In Data Preparation we describe the implementation details related to processing the MEDLINE database for input into CBAG. In Results we present both qualitative and quantitative comparisions between abstracts generated by CBAG, original human-authored abstracts, and abstracts generated by the similar GPT-2 model. In Related Work we discuss the similarities and differences between our proposed method and a collection of contemporary techniques for working with similar textual data. In Future Challenges and Ethical Considerations we discuss the future direction of works like CBAG as well as the ethical implications therein.

### Our contribution

We present CBAG, a transformer-based language model for conditional biomedical abstract generation. Trained using MEDLINE records and informed by semi-supervised domain-specific annotations, this model captures biomedical jargon, entities, and pattern of scientific discussion. We compare this model to two instances of GPT-2, both original and finetuned, and find competitive quantitative results.

All code, data, pre-trained models, preprocessing pipelines, and experimental parameters are available online at https://sybrandt.com/2020/cbag. We additionally supply a set of over 13,000 automatically generated abstracts for a wide range of test set titles. Using the generalizable precondition approach presented here, we hope to enable future applications, such as descriptive hypothesis generation. However, we are also cognisant of the potential for abuse surrounding high quality domain-specific language models. We discuss these concerns further in Future Challenges and Ethical Considerations.

## Background

While recent **language models** receive a newfound popularity in proportion to their surprising capacity across a range of tasks [5], their study predates modern machine learning techniques [15]. Formally, a language model is a probabilistic model that captures the conditional probability of each next element in a sequence given all prior elements. Specifically, this is described by the function:

$$\begin{array}{c} Pr\left(s\right)=\prod_{i=1}^{n}Pr\left(s_{i}|s_{1},\ldots ,s_{i-1}\right). \end{array}$$

Here, *s* is a sequence of *n* elements. The probability of observing sequence *s* is determined by the product of the conditional probabilities of observing each token *s*_*i*_ given all prior tokens. These models can generate new text by iteratively sampling new elements from the probability distribution Pr(*s*_*i*+1_|*s*_1_, …, *s*_*i*_).

The conditional language model introduces a new term *c* into the above equation. The condition can allow applications to alter the resulting sequence based on a priori knowledge [10]. Formally, the conditional language model is defined as:

$$\begin{array}{c} Pr\left(s|c\right)=\prod_{i=1}^{n}Pr\left(s_{i}|s_{1},\ldots ,s_{i-1},c\right)\text{.} \end{array}$$

Modern neural network language models [5, 8] handle these probability distributions by minimizing the negative log-likelihood of these distributions over a large training set of sequences. The loss associated with a dataset of *m* sequences is defined as:

$$\begin{array}{c} L((s^{\left(1\right)},c^{\left(1\right)}),\ldots ,(s^{\left(m\right)},c^{\left(m\right)}))=-\sum_{j=1}^{m}\sum_{i=1}^{n}\log Pr_{\theta }(s_{i}^{\left(j\right)}|s_{1}^{\left(j\right)},\ldots ,s_{i-1}^{\left(j\right)},c^{\left(j\right)}), \end{array}$$

where $s_{i}^{\left(j\right)}$ denotes the *i*^th^ element of sequence *s*^(*j*)^. Here, Pr_*θ*_ indicates the parameterized model that approximates the language model distribution. Modern systems often use the transformer architecture [5, 8, 16] for state-of-the-art quality estimating Pr_*θ*_.

**The transformer** [2], a sequence-to-sequence model built through multi-headed attention layers, has been customized for a number of NLP tasks, as best demonstrated by BERT [3], GPT-2 [5], and a range of notable follow-ups [17–19]. Conceptually, the attention mechanism works by learning multiple weighted averages per-element of the input sequence. Specifically, this includes three projections of each element’s embedding, represented as matrices: *Q*, *K*, and *V*. The rows of each matrix correspond to different projections of the input sequence embeddings. The *Q* matrix acts as a “query” that is compared against “keys” *K* and “values” *V*. The specific mechanism is defined as follows, with *d* representing the dimensionality of each *Q* and *K* embedding:

$$\begin{array}{c} \text{A}\left(Q,K,V\right)=\text{softmax}(\frac{QK^{⊺}}{\sqrt{d}})V. \end{array}$$

The “multi-headed” aspect of the transformer indicates that the attention mechanism is applied multiple times per-layer, per-element of the sequence. These multiple heads, *h*_*i*_, are then recombined through a feed-forward layer. If *X* and *Y* are comprised of row-wise embeddings, and the values $\Theta^{\left(1\right)},\Theta_{i}^{\left(2\right)},\Theta_{i}^{\left(3\right)},\Theta_{i}^{\left(4\right)}$ correspond to four different trainable weight matrices, and all but Θ^(1)^ are associated with the *i*^th^ attention head, then multi-headed attention is defined as:

$$\begin{array}{c} \text{MH}\left(X,Y\right)=\left[h_{1};\ldots ;h_{k}\right]\Theta^{\left(1\right)},\\ \text{where}\;h_{i}=\text{A}(X\Theta_{i}^{\left(2\right)},Y\Theta_{i}^{\left(3\right)},Y\Theta_{i}^{\left(4\right)}). \end{array}$$

The transformer model presented by Vaswani et al. [2] uses the attention mechanism in three different ways. Within the encoder stack, which processes the input sequence in their proposed sequence-to-sequence model, the *K*, *Q*, and *V* embeddings all come from the same sequence of tokens. This is referred to as “self attention.” In the decoder stack, the part of the model that uses the encoder output to generate a new sequence, these embedding matrices are masked during the attention function such that the output embedding for position *i* can only depend on prior elements. This is called “masked self attention”. Following this operation, each decoder embedding is attended with all of the encoder embeddings. Specifically, *Q* values are derived from the decoder, while *K* and *V* values depend on the encoder. We refer to this operation as “Encoder-Decoder Attention.” Note that BERT [16] uses only the encoder self-attention layers, while GPT-2 [5] uses the decoder’s masked self-attention layers. The work presented here uses all three.

The multi-head components are combined with a feed-forward operation, denoted FF, that projects the concatenated embedding into a larger dimensionality, applies the Rectified Linear Unit (ReLU) activation function, and then reduces back to the set embedding rank. Here, Θ^(5)^ and Θ^(6)^ are two new matrices of trainable weights.

$$\begin{array}{c} \text{FF}\left(X\right)=\text{max}\left(0,X\Theta^{\left(5\right)}\right)\Theta^{\left(6\right)}\text{.} \end{array}$$

Then, combined with a learned layer-wise normalization, these components combine to form encoder and decoder blocks. Omitting the standard dropout between each operation, the encoder block is defined as:

$$\begin{array}{cc} E(X) & =\text{LayerNorm}(\text{FF}(\alpha )+\alpha )\\ \alpha & =\text{LayerNorm}(\text{MH}(X,X)+X), \end{array}$$

while the decoder block is defined as:

$$\begin{array}{cc} D(X,Y) & =\text{LayerNorm}(\text{FF}(\alpha )+\alpha )\\ \alpha & =\text{LayerNorm}(\text{MH}(\beta ,Y)+\beta )\\ \beta & =\text{LayerNorm}(\text{MH}(X,X)+X). \end{array}$$

## Multi-conditional language model

**Fig 1:**
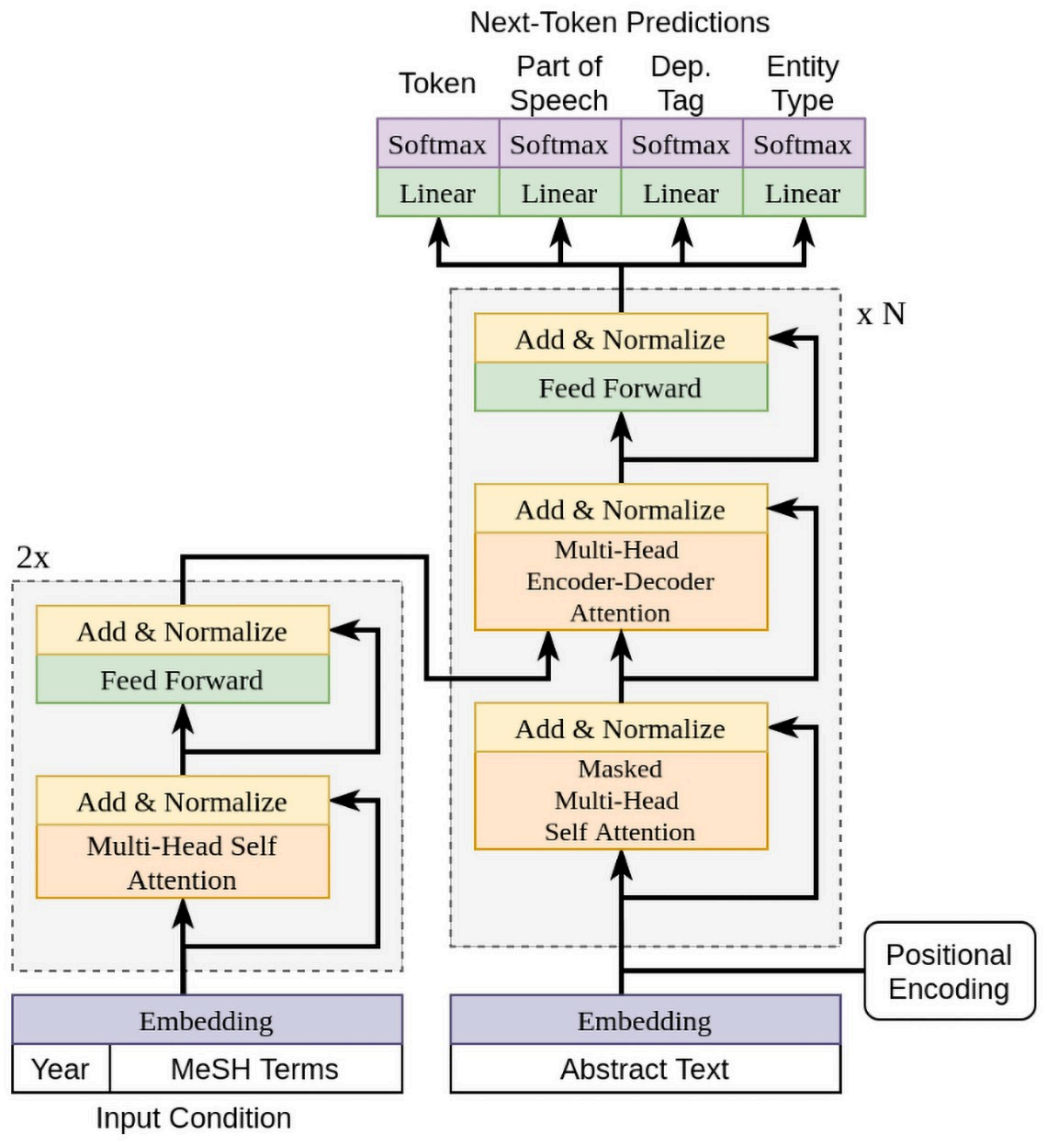
Abstract generator model. Adapted from [2].

The CBAG model follows the transformer architecture [2] with a shallow “condition” encoder, and a deep “language model” decoder. Our adaptation of the transformer model is depicted in Fig 1. The condition is specified as a set of embeddings that enable a high degree of control. To capture information that is particular to language within biomedical domain, we add terms in our objective representing not only elements of the textual sequence, but also the part-of-speech, dependency tags, and entity class labels associated with each textual element. For each class of prediction, we minimize the sum of negative log-likelihood:

$$\begin{array}{c} L\left(t,p,d,e,c\right)=L_{T}\left(t,t,c\right)+L_{P}\left(p,t,c\right)+L_{D}\left(d,t,c\right)+L_{E}\left(e,t,c\right), \end{array}$$

where *t* = *t*_1_, …, *t*_*n*_ are the set of ground-truth textual elements, each with associated *p*_*i*_ ∈ *p* part-of-speech tags, *d*_*i*_ ∈ *d* dependency labels, *e*_*i*_ ∈ *e* entity labels. The term *c* = *c*_1_, …, *c*_*m*_ indicates the set of conditions associated with *t*, and captures information such as metadata keywords and the publication year of the ground truth elements. Each term of L follows the form of:

$$\begin{array}{cc} L_{\left[\cdot \right]}\left(\ell ,t,c\right) & =\sum_{i=1}^{n}-p_{\ell_{i}}^{\left(i\right)}+\log(\sum_{j\neq i}\exp(p_{j}^{\left(i\right)}))\\ \text{where}\;p^{\left(i\right)} & =\text{softmax}(H(\{t_{1},\ldots ,t_{i-1}\},c)\Theta_{\left[\cdot \right]}) \end{array}$$

and the symbol [⋅] is replaced by *T*, *P*, *D*, or *E* for each classification objective. The sequence *ℓ* indicates the ground-truth labels associated with each element of *t* with respect to the particular classification task. Additionally, H(t,c) is the proposed transformer model, which accepts all text elements {*t*_1_, …, *t*_*i*−1_} and *c* in order to produce an encoding for *t*_*i*_. This model is defined as:

$$\begin{array}{cc} H(t,c) & =D_{k}\\ D_{i+1} & =D\left(D_{i},E_{l}\right)\;\text{and}\;D_{0}=t+\text{PE}\\ E_{i+1} & =E\left(E_{i}\right)\;\text{and}\;E_{0}=c. \end{array}$$

Each input element of *t* and *c* is first assigned an input encoding and put through their respective stacks of encoder and decoder layers. Additionally, *k* and *l* refer to the number of decoder and encoder layers respectively. The symbol PE references the positional encoding defined by the sinusoidal function presented in [2]. This encoding enables the transformer model to take position into account when considering embeddings, and is defined as:

$$\begin{array}{cc} PE_{pos,2i} & =sin(pos/10000^{\frac{2i}{d}})\\ PE_{pos,2i+1} & =cos(pos/10000^{\frac{2i}{d}}), \end{array}$$

where *pos* indicates the embedding’s position in the input sequence, and *i* denotes the dimension along each size-*d* embedding.

Initial input encodings are provided by an embedding table that begins randomly initialized. We determine textual elements through the unigram word-part tokenizer [12], and contextual elements consist of a learned embedding per-publication year, as well as embeddings for each Medical Subject Heading (MeSH term).

### Hyperparameters

We selected hyperparameters similar to the GPT-2 “medium” model. This includes an embedding dimensionality of *d*_*k*_ = 1024, *k* = 16 attention heads per multi-headed attention layer, *e* = 2 encoder blocks, *d* = 16 decoder blocks, a fully-connected size of 3072, and an inner-block dropout rate of 0.1. We additionally use a max sequence length of *n* = 128. Our set of initial embeddings contains 16,000 text tokens, 48,133 MeSH headings, and 230 year embeddings.

### Optimization

We minimize L using the large-batch optimizer LAMB [20] across 40 Nvidia V100 GPUs using an effective batch size of 480. We selected a learning rate of 0.001, with a 500-batch linear warm up. We check pointed the model each epoch after viewing 5% of the training data (about 700,000 abstracts). Note that each time an abstract is viewed, we select from it a different training window. We trained this model for 72 hours using PyTorch Lightning [21] to aid in the distribution and check pointing.

## Data preparation

**Fig 2:**
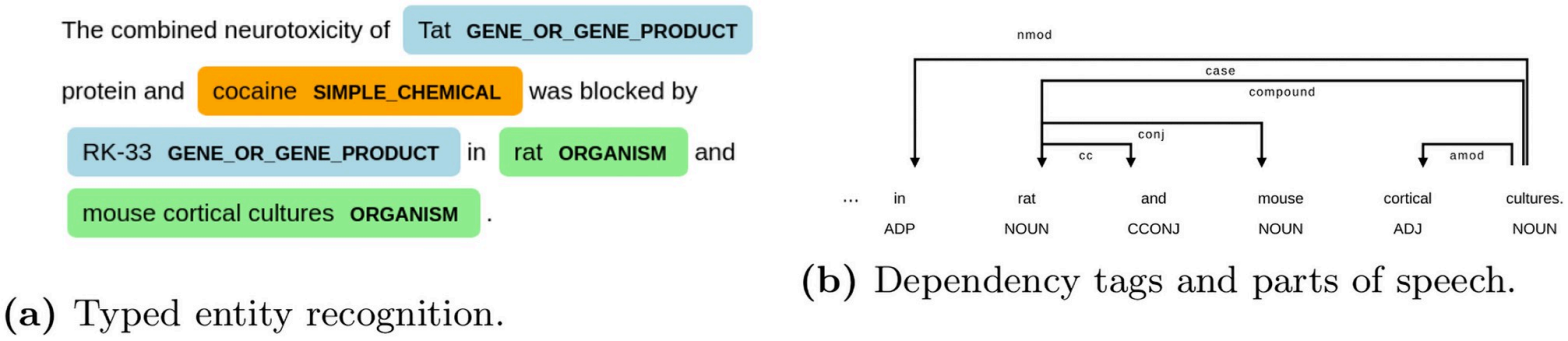
Annotations provided by ScispaCy “BIONLP13CG”. **(a)** Typed entity recognition. **(b)** Dependency tags and parts of speech.

In order to train the model described in Multi-Conditional Language Model, we collect training samples (*t*, *c*) from the set of publicly available biomedical abstracts provided in the MEDLINE database. This dataset contains publication dates, author-supplied MeSH terms, titles, and abstracts for more than 30-million citations. We filter for documents that were originally published in English, as well as documents that contain at least one non-title sentence. Documents without metadata keywords are allowed. We split the remaining abstracts into a training and test sets following a 70–30 split.

Within the domain of biomedical text mining, there are relatively few annotated training sources [11, 22]. To endow the CBAG model with biomedical-domain knowledge, we automatically annotate the entire MEDLINE training set using the ScispaCy model [23] trained on the “BIONLP13CG” BioCreative dataset [11]. We selected this particular model because it produces the widest range of entity labels when performing named entity recognition. These consist of: cancer, organ, tissue, organism, cell, amino acid, gene or gene product, simple chemical, anatomical system, immaterial anatomical entity, multi-tissue structure, developing anatomical structure, organism subdivision, and cellular component. We add a class corresponding to “not an entity” as well.

Using the ScispaCy model and a cluster of 100 machines, we quickly identify every token, part-of-speech, dependency tag, and entity label for all 14-million training-set MEDLINE documents. We depict examples of these automatic annotations in Fig 2. However, in order to formulate these textual features for input into the CBAG model, we also leverage the unigram subword regularization method from Kudo et al. [12]. This method learns an efficient tokenization sentences. Each token corresponds to a “chunk” of characters, many of which correspond to subword components. The unigram approach adds a normalization factor wherein the specific tokenization for each word is probabilistic determined from the set of ambiguous subword sequences. These subword sequences, along with special “start of abstract” and “end of abstract” tokens, create input *t*.

We train the unigram tokenization method on one-million randomly sampled sentences from the training set, specifying a fixed-size vocabulary of 16,000 subword tokens. We additionally lowercase the entire training corpus, and enforce that every character within the sampled training set receive its own token. Using the resulting model, we tokenize the entire training set, and cross reference the subwords with the multi-task labels provided by ScispaCy. This way, each subword token *t*_*i*_ in the training set is associated with a part-of-speech *p*_*i*_, dependency tag *d*_*i*_, and entity label *e*_*i*_.

Next we index each training-set publication years and author-supplied MeSH keywords, which form the condition *c*. For publication years, we simply identify the earliest year within the training set, 1790, and add an index for each year between then and 2020. We identify over 4-million author-supplied keywords within MEDLINE, which is prohibitively large for our model to capture. We prune any keyword that occurs fewer than ten times, reducing that set to a manageable 48,133. We add each to our excising embedding index, which contains nearly 50,000 total embeddings.

When training, we select a batch of abstracts, and for each abstract we select a window of 128 subword tokens to form *t*, restricted such that the first token of each window corresponds to the first token of a sentence. In addition, we supply the condition indices *c*. The sequence of labels *ℓ* is formulated by shifting the subword token window by one token, such that *t*_*i*−1_ is used to predict *t*_*i*_, *p*_*i*_, *d*_*i*_, and *e*_*i*_.

**Fig 3:**
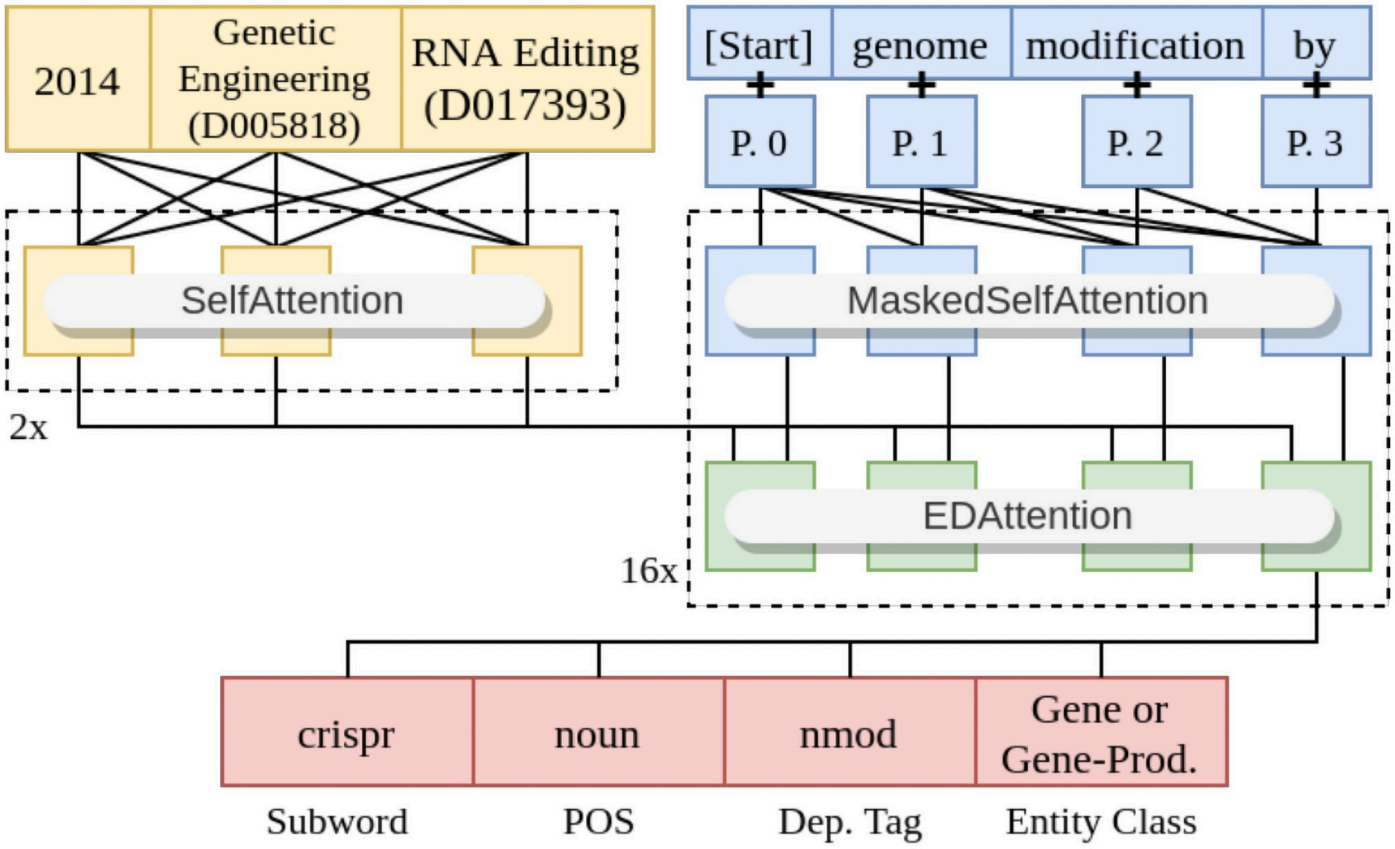
Abstract generator example input.

An example of model input and output is depicted in Fig 3.

## Results

While NLP benchmarks such as GLUE [24] and its biomedical counterpart BLUE [22] help researchers compare performance across a range tasks, we are unaware of a benchmark for the generation of biomedical abstracts. In lieu of such a dataset, we leverage our held-out test set of Medline abstracts, and a set of traditional NLG metrics [14]. We generate abstracts by providing a title *t* and condition *c* from a test set abstract. We extend *t* by sampling from the resulting probability distribution over subword tokens *p*^(*i*)^ until observing the “end of abstract” special token, or until the generated text has reached a pre-determined maximum token length. We then compute, Bleu [25], METEOR [26], ROUGE-L [27], and CIDEr [28], by comparing each generated sentence against the set of “reference” sentences comprising the corresponding human-written abstract.

To add context to our reported performance numbers, we also generate text using OpenAI’s recently released 1.5-billion parameter “huge” GPT-2 model [5]. This model has been shown to excel on a number of tasks without modification, inducing as a replacement to traditional knowledge bases [13]. However, as this model was trained to generate language found online, such as in the BooksCorpus and English Wikipedia, it is at a disadvantage when generating domain-specific text. Because GPT-2 does not produce any “end of document” indicator, we generate the same number of subword tokens as present in the human-written counterpart, and truncate the potential partial sentence at the end of the abstract.

**Table 1:** (Part I) full abstracts generated with respect to the same title.

**(PMID: 28026780) Hierarchically Micro- and Nanopatterned Topographical Cues for Modulation of Cellular Structure and Function.**
**Original Author’s Abstract**
Living cells receive biochemical and physical information from the surrounding microenvironment and respond to this information. Multiscale hierarchical substrates with micro- and nanogrooves have been shown to mimic the native extracellular matrix (ECM) better than conventional nanopatterned substrates; therefore, substrates with hierarchical topographical cues are considered suitable for investigating the role of physical factors in tissue functions. In this study, precisely controllable, multiscale hierarchical substrates that could mimic the micro- and nanotopography of complex ECMs were fabricated and used to culture various cell types, including fibroblasts, endothelial cells, osteoblasts, and human mesenchymal stem cells. These substrates had both microscale wrinkles and nanoscale patterns and enhanced the alignment and elongation of all the cells tested. In particular, the nanotopography on the microscale wrinkles promoted not only the adhesion, but also the functions of the cells. These findings suggest that the hierarchical multiscale substrates effectively regulated cellular structure and functions and that they can be used as a platform for tissue engineering and regenerative medicine.
**CBAG**
the ability to integrate multiple physiological cues and thereby mediate many cellular functions is critical for many complex life history processes. despite recent advances in high-throughput imaging of biomolecules and their spatiotemporal integration into dynamic structure and function, the precise structural organization and temporal structure of tissue architecture remains poorly understood. here, we present an efficient system for temporally and spatially mapping micro- and nanopatterned topographical cues in organ-specific spatial and temporal properties using multiple imaging modalities. the micro-/nanopatterned geometrical cues can be localized to cell membranes, cells, and proteins. the spatial and temporal dynamics of these local signals are precisely represented by the cross-correlation function, which forms the basis of a geometrical model that accurately provides spatiotemporal information about the spatial location and spatial coordinate of the labels and their functionalities. the model is also capable of correlating the properties of neural cells within their network without affecting the spatial and temporal organization of their spatial features, as well as those of their surrounding tissue. as an example of this model, cell types grown as multilayers are described.

**Table 2:** (Part II) full abstracts generated with respect to the same title.

**(PMID: 28026780) Hierarchically Micro- and Nanopatterned Topographical Cues for Modulation of Cellular Structure and Function.**
**GPT-2 Huge Unmodified**
[\n][\n] The first example of what happens when an artificial neural network’see’ complex features on a computer screen is provided by the vision of people with sight loss. The most recent results (e.g., see Krueger’s blog) suggest that a person who has a damaged retinal pathway for visual light-scattering called LID is able to’see’ objects using low power laser sources. The finding is unexpected because it suggests an adaptive mechanism for recovering limited visual function. The finding has profound implications for understanding how the retina and brain develop, how vision is learned, and how people can potentially regain impaired vision with new retinal implants. [\n] The retinotopic map shows which parts of the retina belong to what parts of the visual field (more details in the video below). [\n] The following images are from a retinotopic map showing a person who has lost some peripheral vision due to an injury in his optic nerve and retinal pigment epithelium. Note that he can make out details when looking toward the left, and when looking toward the right, details become distorted or are obscured.
**GPT-2 Small Finetuned**
The development of new materials for biomedical applications is a major challenge. Here, we report a novel approach to the fabrication of hierarchical microand nanostructures for the modulation of cellular structure and function. The hierarchical micro- and nanostructures are fabricated by a simple one-pot process using a simple one-pot method. The hierarchical micro- and nanostructures are fabricated by a simple one-pot method using a simple one-pot method. The hierarchical micro- and nanostructures are fabricated by a hybridize the nanostructures are fabricated by a hybridize the nanostructures are fabricated by a two-based on the nanostructures. The nanostructures are fabricated by a nanostructures. The nanostructures are fabricated by a nanostructures are fabricated by a nanostructures are fabricated by a nanostructures are fabricated by a nanostructures. The nanostructures are fabricated by a nanostructures are fabricated by a nanostructures are fabricated by a nanostructures.

We present a full abstract from both CBAG and GPT-2 in Tables 1 and 2. Note, newline characters produced by GPT-2 are replaced with “[\n]” due to space limitations. In this example, we observe that the CBAG model recovers a set of relevant biomedical entities. Unsurprisingly, the model parrots some entities that appear in the title, such as, “micro- and nanopatterned topographical cues,” as well as “cellular functions” in this example. However, it is also able to produce more advanced concepts including “multiple imaging modalities,” and “multiscale substrates” that do not appear in the title but do appear in the corresponding human-written abstract (not reproduced here for space concerns, but is publicly available). The GPT-2 model does recover some biomedical entities, such as “damaged retinal pathway” and “retinal pigment epithelium,” however these keywords are unrelated to the considered document. Other out-of-context entities such as “artificial neural network,” “computer screen,” and reference to a blog reduce the ability of a human reader to extract any meaningful biomedical information from this text. We find that these example abstracts help motivate the need for domain-specific language models.

**Table 3:** Differing generations of the same prompt given various MeSH preconditions. We record the first sentence completing the prompt *“In this study, we found…”*.

Condition	CBAG’s Response
D003270: Contraceptive Agents	…that, during a prospective observational period, the patients were aware of the possibility of adverse cardiac events.
D003634: DDT	…that the aromatic (g)-tse, which is often produced in fruit, is potentially useful to suppress green algae as well as pesticide toxicity.
D004042: Unsaturated Dietary Fats	…that vitamin e levels are associated with early childhood health consequences.
D006046: Gold	…that the nanoparticles provide improved sensitivity to gold nanoparticles, and they are sensitive to ag-b interaction rather than ca-a interaction.
D005395: Fish Oils	…that the combination of pinkland and fish oil intakes (ca-like and ca-like) improves the antioxidant effect of yinneria (tricapsa vul) and that can significantly decrease food intake.

Because CBAG is a conditional language model, we explore the range of responses the model can produce given different conditions. In Table 3 we present the first sentence produced by the model for the input “*In this study, we found…*” given different conditions. The results indicate that the condition has a significant impact in the resulting text. When conditioned with the MeSH term for contraceptive agents, the model discusses a patient study on cardiac side-effects. The output conditioned on the pesticide DDT describes fruit and toxicity. The output on gold describes gold-nanoparticle sensitivity. These results demonstrate the ability for the CBAG model to learn domain-specific research content provided by various keyword preconditions.

**Table 4:** CBAG (left) compared to GPT-2 huge unmodified with 1.5B parameters (right). Both systems are given the same title as a prompt. CBAG receives metadata. Results truncated for space.

**(PMID: 28029317) Laparoscopy to Predict the Result of Primary Cytoreductive Surgery in Patients With Advanced Ovarian Cancer: A Randomized Controlled Trial.**	
laparoscopic surgery is the standard treatment for patients with advanced ovarian cancer; however, these patients do not receive a standard palliative regimen.	J Natl Cancer Inst 2008;100:1567–1572. 24. The focus of this review is the effect of apoE4 levels on the risk of poor surgical outcome in patients with advanced ovarian cancer.
**(PMID: 27993387) Low vitamin D does not predict statin associated muscle symptoms but is associated with transient increases in muscle damage and pain.**	
in clinical practice, patients with moderate-to-severe hypervitaminosis d present with debilitating side effects related to statin use.	ow vitamin d does not predict statin associated muscle symptoms but is associated with transient increases in muscle damage and pain.
**(PMID: 28012718) Skin-Resident Effector Memory CD8+CD284− T Cells Exhibit a Profibrotic Phenotype in Patients with Systemic Sclerosis.**	
systemic sclerosis (ssc) is an inflammatory disease characterized by the infiltration of t cells into skin and skin surfaces. the presence of autoantibodies can lead to the development of cutaneous t-cell hyperactivity.	J. Clin. Invest. 117: 2748–2759; Dilating collagen in chronic neuropathic pain. Arch. Neurol. 63: 983–989
**(PMID: 27999935) Laparoscopic sentinel node navigation surgery for early gastric cancer: a prospective multicenter trial.**	
to compare the feasibility and safety of laparoscopic sentinel node navigation surgery with that of conventional in-field navigation (oif) surgery in the treatment of early gastric cancer (egc).	Patel S et al. (2003) Age associated factors associated with false-positive result of prognostic biomarkers in prostate and breast cancer.

To provide further qualitative comparison between the considered models, we additionally provide a few first-sentences produced given various test set titles in Table 4. In these sentences, and across the test set, we observe that CBAG produces a number of scientific clichés. Most clearly, the model captures biomedical turns of phrase such as “in clinical practice.” Additionally we observe that it is common for CBAG to produce an entity followed by an abbreviation that it will repeat throughout the text. However, we observe that some abbreviations are not sensible from a human perspective, such as “in-field navigation (oif).” In these cases, the incorrect abbreviation will still be repeated by the model.

Not seen in these first-sentences is a trend for the model to follow major abstract claims with a fictional *p*-value or sample-size. We find *p*-values in approximately 10% of abstracts, with a median value of 0.02, and when plotting this distribution of generated *p*-values we find it matches the expected (and troubling) trend of *p*-values in real-world science [29]. Additional examples of these trends can be found in our supplemental data online https://sybrandt.com/2020/cbag.

### Quantitative analysis

In order to understand quantitative relationships between each model, we turn to a set of NLG metrics, which are nicely summarized by [14]. At a high level, each metric rewards models that generate text that is similar to that produced by human authors for the same prompt. Each metric would then assign a numeric value to our model’s generated text based on whether it shared similar characteristics with any of the human-provided responses. In our case, we adapt these metrics for scientific text. We consider an article’s title to be the “prompt” and we consider the body of the abstract to be a single “response” that our algorithm will be judged against.

The different metrics we consider each value different textual characteristics. The simplest is the Bleu metric [25], which rewards models that generate *n-grams* that are present in the reference text. An n-gram is simply a series of *n* words in a specific order. The word “Hello” would be a 1-gram, while “my name is” would be a 3-gram. Typically it is infeasible to consider all n-grams of a given text, so instead Bleu is typically restricted to a range of *n* values. Specifically, if we wanted to compute Bleu_*k*_ (the Bleu value considering only *k*-grams) for a generated hypothesis text *H* with respect to a single reference text *R*, we would compute:

$$\begin{array}{c} {\text{Bleu}}_{k}={\left|G_{k}\right|}^{-1}\sum_{G_{k}}\min(\text{Count}\left(G_{k},H\right),\text{Count}\left(G_{k},R\right)) \end{array}$$

where *G*_*k*_ is the set of all *k*-grams in *H*, and Count(*G*_*k*_, *X*) produces the number of occurrences of *G*_*k*_ in text *X*.

In this work we consider Bleu_1_, as well as Bleu_1+2+3+4_, which sums the Bleu-scores for 1–4 grams. We also consider similar metrics such as METEOR [26], CIDEr [28], and ROUGE-L [27]. METEOR is similar to Bleu, but first applies stemming, synonym matching, and imposes a constraint regarding the ordering of n-grams. CIDEr weights Bleu-scores by the Term-Frequency Inverse-Document-Frequency (TF-IDF) of n-grams in the reference. ROUGE-L is a slightly different metric in that it considers only the longest common subsequence shared between the hypothesis and reference. Specifically, if *S* is the longest common subsequence shared between *H* and *R*, then ROUGE-L is defined as:

$$\begin{array}{cc} p & =\frac{\text{Length}(S)}{\text{Length}(H)}\\ r & =\frac{\text{Length}(S)}{\text{Length}(R)}\\ \text{ROUGE-L} & =\frac{(1+\beta^{2})pr}{r+\beta^{2}p} \end{array}$$

where *β* is a constant that balances the tradeoff between *p* and *r* (typically, *β* = 1.2).

**Fig 4:**
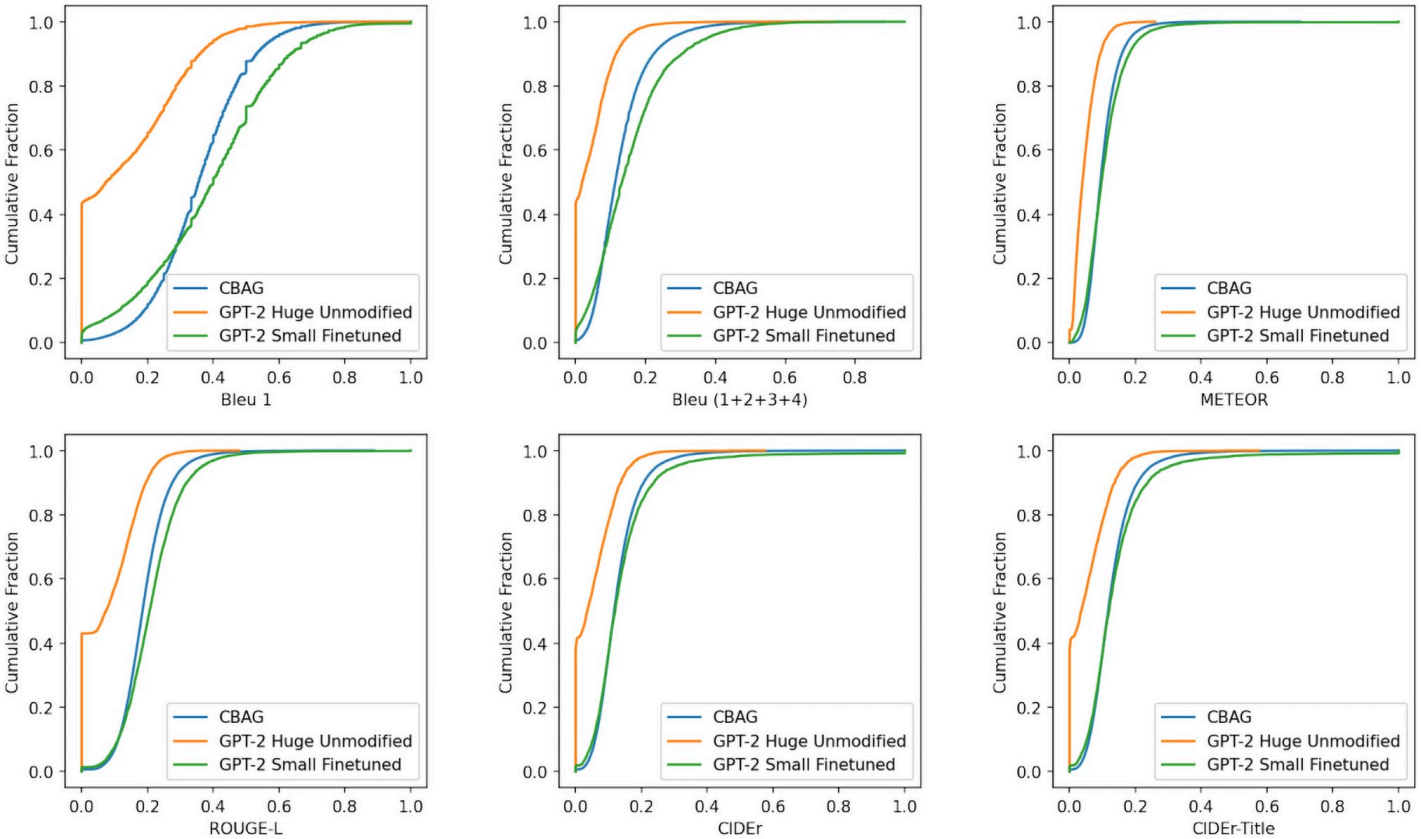
Cumulative score distributions comparing GPT-2 huge unmodified, GPT-2 small finetuned, and CBAG. Higher values for all considered metrics are better. If a model achieves higher metric scores across a wider set of examples, the resulting cumulative score distribution will have a *lower* area under the curve.

In addition, we compute a customized “CIDER-Title” metric that sets the weight of any *n*-gram that appeared in the title to zero. This quantified the ability of each model to produce non-trivial *n*-grams in the abstract body. The cumulative sentence-wise score distribution for all metrics for a sample of test set abstracts are depicted in Fig 4.

We find that both the CBAG and the GPT-2 Small Finetuned models perform almost identically in *n*-gram recall. The GPT-2 Huge Unmodified model, while capable of producing some sentences that score highly on these recall-oriented metrics, does so less frequently. Note that the above plots, being sentence-wise metrics, are unable to penalize a model for producing repeated sentences. For instance, one GPT-2 Small Finetuned generation produces the correct rare phrase “phosphatidylinositol” repeatedly, resulting in a high METEOR and Bleu-1 score. As a result, we find the above quantitative measures demonstrate similar keyword recall capabilities of both CBAG and GPT-2 Small Finetuned, even if they can produce qualitatively different results. This finding emphasizes the need for new metrics when comparing text generation results with only single reference examples.

## Related work

**BERT** [3] is a transformer-based model that consists a stack of unmasked multi-headed self-attention, which means that every output embedding depends on all input embeddings. This all-to-all dependency is what the authors mean when describing the model as “bidirectional,” which departs from the more traditional left-to-right, right-to-left LSTM model.

When training BERT, input text is tokenized by the WordPiece algorithm [30], and two different types of training examples are input. In the first, some tokens are randomly replaced with a masked reserve token. The objective of the model during the unsupervised pre-training phase is to predict the original token, using the rest of the input. In the second, two sentences are supplied and, using the output embedding of the “start-of-input” character, the model must determine whether the second sentence followed the first in the training data.

**GPT** [4] and **GPT-2** [5] both use a transformer-decoder stack of *masked* multi-headed self-attention. The mask, in this case, enforces that the output embedding of token *i* may only depend on inputs 1, …, *i*). This masking formulation, which we adopt in this work, restricts the GPT-models to function as pure language models. These models are pre-trained through a generative objective. For each input sequence 1, ⋯, *n*, the model is input 1, ⋯, (*n* − 1) and required to generate the sequence 2, ⋯, *n*. Due to the masked-self-attention layers, this means that each prefix sequence of the input is simultaneously predicted each follow-up word.

The major difference between the GPT and GPT-2 models is the larger training corpus, which leads to state-of-the-art text generation. In [5], this model is even shown to improve the state-of-the-art of other objectives such as question answering and translation, even without a finetuning phase. Follow-up work [13] identifies that high-performance language models like GPT-2 can even replace specialty knowledge-bases.

**XLNet** [31] modifies some of the assumptions underpinning the BERT model in order to improve the pre-training of its Transformer-XL [32] inspired architecture. While the BERT model pre-trains be predicting masked tokens from input sequences, XLNet learns the probability distribution of tokens based on the set of permutations in the factorization order of pre-training sequences. As a result, XLNet can learn to use both forward and backward context without introducing a mask token that is only used in pre-training. As a result this technique outperforms BERT across a range of natural language understanding tasks. However, the authors comment that the benefits of this technique come from the ability to better leverage forward and *backward* context, which makes this technique hard to apply to text generation, where backward context is not available.

**Performers** [33] are models that may provide a more efficient alternative to the self-attention mechanism of the transformer architecture without compromising on modeling quality. Using the Fast Attention Via positive Orthogonal Random features (FAVOR+) approach, that provides a provably accurate estimate of the transformer self-attention mechanism, using linear time and space. A modification of this approach, also in the initial Performer paper, uses a prefix-sum operation to estimate forward-directional attention in a manner that would be compatible with language modeling. As a result, it is likely that language generation models will be able to use this technique to reduce the computational costs associated with today’s state-of-the-art models. While this is an important step to making language model’s more applicable to everyday tasks, the downstream performance of both neural architectures is very comparable.

**SciBert** [6] achieves state-of-the-art performance across a range of scientific NLP benchmarks by retraining the WordPeice tokenizer [30], and a BERT model [3] on 1.14-million papers collected by semantic scholar. In [6], Beltagy et al. demonstrate that by performing unsupervised pre-training on this scientific dataset, they are able to improve performance over the standard BERT-pre-trained weights on their ultimate finetuned models for entity recognition, PICO extraction, text classification, relation classification, and dependency parsing. These findings make the case that scientific text is sufficiently dissimilar from that found in general language to require custom models.

**BioBert** [7] follows the same pattern as SciBert, but pre-trains on the biomedical texts supplied by MEDLINE and PubMedCentral. As opposed to SciBert, this method does not replace the general-language training data supplied by English Wikipedia and BooksCorpus, and instead appends both biomedical text databases. In [7] Lee et al. explore the resulting finetuned performance across a large range of small biomedical NLP tasks, and find mixed results. We interpret these results to indicate the importance of finding training data that is not only sufficiently large, but also relevant to the task at hand.

**Covid-twitter-BERT** (CT-BERT) [34] is a pretrained BERT model that was trained to model tweets pertaining to the COVID-19 pandemic. This model demonstrates the ability for NLP models of all varieties to specialize for different applications, and that one can expect a significant improvement in task-specific tasks if one first derives a task-specific model. In this work, CT-BERT outperforms the BERT-Large model by an average of 17.57% across five NLP tasks. In this work we see a similar behavior wherein a biomedically specialized model can provide significant advantages over a more general NLP solution. As discussed for other BERT-based models, the limitations of BERT for NLG remain a reason to consider specialized NLG-specific architectures when generating scientific text.

**Wang et al.** [16] explore the capacity for a BERT model to effectively function as a Markov random field language model. This technique takes advantage of the masked pre-training used in the base BERT model to predict unknown tokens. This approach also departs from the traditional language model described here as every sequence element determines the probability of every other element. Generation is performed by iterative freezing highest-probability elements from within a fixed-length sequence of initially free variables.

**CTRL** [8] is a conditional language generation method that extends GPT by including “control codes” that prefix the sequence of text elements. For instance, each website represented in the training data is represented by a code, and as a result generated text can switch styles based on these prefixes. Additionally, various model functions, such as question answering, are learned via generation with various codes. As a result, prefixing questions with the respective code results in a higher probability assigned to relevant answers. Furthermore, this work includes some multi-code prefixes, such as “Rating 5.0” or “Sentence Title” to further condition the generated result. While the CTRL model is the most similar to the method presented here, it has some key differences. Firstly, the CTRL model uses prefix tokens to condition generated text, while we apply a shallow transformer-encoder stack. As a result, the CTRL approach is limited in that training requires a strict set of codes, or a small set of enumerable code-pairs. In contrast, the CBAG approach allows the method proposed here to accept arbitrary-length sequences of keywords as a condition.

## Future challenges and ethical considerations

Many readers have likely heard of paper generators similar to Scigen [35]. This particular project generates computer science full-text articles by randomly sampling from a context-free grammar, and has produced publications actually accepted by some venues. This 15-year-old system, however, is incapable of fooling but the least-observant of gate keepers. However, high quality text generation introduces NLP to a range of challenges currently posed by “deepfake” images. These problematic pictures permeate the zeitgeist and stir a response reaching further than computer science [36], extending into law [37], culture [38], and philosophy [39]. Meanwhile, misinformation spread by human actors online already cascades throughout social network echo chambers at an alarming rate [40]. One needs very little imagination to conceive of ways that the automatic generation of “pseudo-science” online could lead to public distrust of the scientific community.

OpenAI is forming partnerships between computer-science and the social sciences in order to understand these implications in society [41]. One major challenge they note is a distinct lack of “correctness” measures for text generation. In completing this work, we find that some correctness measures do exist, such as the SPICE metric to judge image caption correctness [42]. Unfortunately, this technique does not scale well to large knowledge bases as it requires the graph of predicate arguments induced by reference sentences. Not only are there a lack of methods to extract arguments from text, but we need to find new algorithms for quantifying correctness for large graphs induced by all of biomedical science.

Despite the potential for abuse, we designed CBAG with our own vision toward enabling human-understandable hypothesis generation systems [43, 44]. For instance, our model architecture could be conditioned on more generalized forms of existing biomedical knowledge, such as semantic graph embeddings, in order to produce textual descriptions of plausible future research directions. These explanations could potentially persuade domain scientists to pursue new research directions, as similar systems have already done [45, 46]. However, these systems require specialized analysis and introduce new cognitive burdens for scientists to understand and act on their outputs. If similar hypothesis generation systems instead could produce human-readable arguments, then we could better utilize the wealth of publicly available information, improve the productivity of biomedical researchers, and ultimately find new treatments and cures for people worldwide.

## Conclusions

We present the Conditioned Biomedical Abstract Generation (CBAG) model for understanding scientific abstracts. We train this model using publicly available biomedical data provide through MEDLINE to predict text that is conditioned on publication year and arbitrary sets of author-supplied keywords. This model leverages the transformer architecture [2], featuring a shallow condition encoder, as well as a deep language model decoder. Across a range of NLG metrics [14], we demonstrate competitive performance with a finetuned version of GPT-2, having only trained on biomedical abstracts. Qualitatively, we present generated sentences and documents that exemplify the sort of quality and control that the CBAG model enables.

We anticipate that conditioned language generation can be used to build new applications in the biomedical domain, such as a hypothesis generation system that produces textual descriptions of proposed new research directions. To do so, the conditional aspect of the CBAG model will likely be a necessity. However, we also acknowledge the ethical considerations behind the proliferation of convincing scientific language generation models. We provide the pre-trained model, more than 13,000 generated abstracts, and all necessary training and evaluation code to aid in exploration and reproducibility.
